# Methanol-to-Olefins Studied by UV Raman Spectroscopy as Compared to Visible Wavelength: Capitalization on Resonance Enhancement

**DOI:** 10.1021/acs.jpclett.4c00865

**Published:** 2024-06-25

**Authors:** Emma Campbell, Igor V. Sazanovich, Michael Towrie, Michael J. Watson, Ines Lezcano-Gonzalez, Andrew M. Beale

**Affiliations:** † Cardiff Catalysis Institute School of Chemistry, Cardiff University, Cardiff CF10 3AT, U.K.; ‡ Department of Chemistry, University College London, 20 Gordon Street, London WC1H 0AJ, U.K.; § Research Complex at Harwell (RCaH), Harwell, Didcot, Oxfordshire OX11 0FA, U.K.; ∥ Central Laser Facility, Research Complex at Harwell, Rutherford Appleton Laboratories, Harwell Campus, Didcot OX11 0QX, U.K.; ⊥ Johnson Matthey Technology Centre, P O Box 1, Belasis Avenue, Billingham TS23 1LB, U.K.

## Abstract

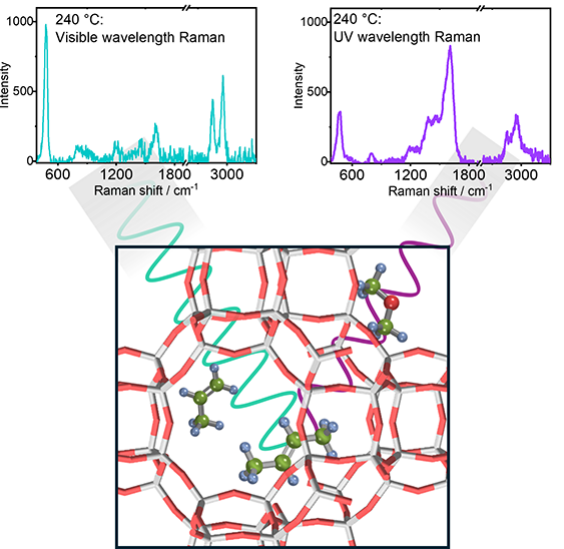

Resonance Raman spectroscopy can provide insights into complex reaction mechanisms by selectively enhancing the signals of specific molecular species. In this work, we demonstrate that, by changing the excitation wavelength, Raman bands of different intermediates in the methanol-to-hydrocarbons reactions can be identified. We show in particular how UV excitation enhances signals from short-chain olefins and cyclopentadienyl cations during the induction period, while visible excitation better detects later-stage aromatics. However, visible excitation is prone to fluorescence that can obscure Raman signals, and hence, we show how fast fluorescence rejection techniques like Kerr gating are necessary for extracting useful information from visible excitation measurements.

The application of Raman spectroscopy to study catalytic reactions is understood to be a challenge due to strong backgrounds that drown Raman signals, often attributed to fluorescence;^1−3^ nonetheless, it can be a powerful tool for understanding catalytic behavior.^1,3−5^ Efforts to avoid fluorescence include using Raman probes of wavelengths in the UV or infrared regions, since fluorescence is typically a problem for the visible-spectral range.^2−6^ It is well-established that, for nonresonant Raman, the probability of Raman scattering is directly proportional to the fourth power of the frequency of light, meaning that infrared excitation typically results in low intensity signals due to the poorer scattering at longer wavelengths, while UV excitation on the other hand generally leads to increased signal intensity across all Raman-active vibrations.^4−6^ Changing the wavelength of laser excitation can cause significant changes in the relative intensities of Raman signals due to the resonance effect, where different chromophores in the molecules absorb at different energies.^1,5,7−10^ When a sample contains an electronic transition near the laser excitation wavelength, scattering is enhanced by up to 10^6^, and this type of Raman spectroscopy is known as resonance Raman spectroscopy.^1,5,7,8^

Resonance Raman (RR) has been demonstrated in the field of catalysis, as an example, by Chua et al., who studied supported metal oxides, where the authors were able to observe changes in the relative intensity of M–O–M vibrations vs M=O, depending on the use of visible or UV wavelength for excitation.^7^ It has also been well-applied in the study of amorphous and/or graphitic carbon deposits, which can help to understand the structure of coke,^9−11^ and in characterizing metal ions in zeolite frameworks.^3,12,13^ Further details and examples of RR can be found elsewhere.^3−5^ In the case of a mixture of chemicals or species, RR may be used to identify individual components in the mixture through selectively probing specific absorption bands.^3,4,7,12^ It is therefore important to consider that through probing a complex catalytic system such as in the hydrocarbon pool mechanism of the methanol-to-hydrocarbon reaction (MTH), by changing the wavelength of excitation, Raman bands of different species can be identified, giving a fuller picture of the mechanism at play. UV–vis spectroscopy has been used as a technique to directly probe the reaction mechanism and shows distinct absorbance bands relating to certain intermediates in different stages of the reaction.^14−16^ The results overall indicate that polyaromatic hydrocarbons alone would be prone to resonance Raman enhancement at IR probe wavelengths, while only a UV probe might be in resonance with the species present during the induction period. Table S1 given in the SI summarizes some absorbance values of such species as involved here, as reported in the literature.^14,16−21^

MTH is a widely studied reaction^22,23^ that can upgrade C_1_ reactant (methanol) to highly valuable olefins or gasoline-grade products over zeolite catalysts via a complicated indirect mechanism known as the hydrocarbon pool.^24^ The reaction products are typically small C_2_–C_4_ olefins, since only small products elute from the pores.^23^ Early kinetic studies showed that the reaction rate increases after an initial induction period,^24^ where larger, bulky hydrocarbon intermediates build up inside the pore system of the zeolite, which are then responsible for catalyzing the formation of olefinic products.^24,25^ The reaction is therefore autocatalytic, and this mechanism is termed the hydrocarbon pool mechanism.^23,24^ The active hydrocarbon pool species vary depending on the zeolite employed for the reaction and play an important role in product distribution.^22,26^

In small-pore zeolites and zeotypes with the chabazite topology (SSZ-13 or SAPO-34), the typical hydrocarbon pool species include olefins, methylated benzenium ions, and naphthalenic hydrocarbons, which undergo repeated methylation by methanol during reaction.^27−29^ According to UV–vis data reported, these species show absorbance maxima around 200–240, 360–390, and 410–430 nm, respectively.^14−16^ Studies have long since shown that in deactivated chabazite catalysts, polyaromatic hydrocarbons are formed inside the zeolite, containing up to four aromatic rings.^14,30^ Where CHA cages contain polyaromatic hydrocarbons consisting of three of four rings, the cages are rendered inactive for MTH.^30,31^

One popular method for characterizing deactivated MTH catalysts is the dissolution of the catalyst framework to extract the hydrocarbons, and a plethora of polyaromatic hydrocarbons have been identified this way in the reaction.^22,32,33^ There is, however, significant advantage to the application of spectroscopy that can be used to characterize the material without its destruction, such as the possibility to characterize under reaction conditions and in their state in/on the zeolite. Using Raman spectroscopy, it is possible to capitalize on resonance enhancement to detect species even in low concentrations, and as previously discussed, use of UV excitation allows the common issue of sample emission to be somewhat minimized.^4^ Signorile et al. put forward several papers using UV Raman spectroscopy to characterize polyaromatic hydrocarbons and identify those formed during MTH,^11,22,26,34,35^ whereas Chua and Stair used UV Raman to study the hydrocarbons retained in H-ZSM-5 after dosing with methanol or dimethyl ether (DME) and flushing with inert gas.^36^ The studies led to the identification of various species in the reaction by their Raman vibrations, including polyaromatic hydrocarbons, conjugated olefins, and cyclopentadienyl species.^22,26,34,36^ Li et al. used UV Raman to study coke formation in methanol dehydration (to DME), and they were able to detect methylbenzenium ions by Raman as “soft-coke” species in the reaction on H-ZSM-5 as a catalyst under *operando* conditions.^37^ A potential downside when using a UV source is an increased risk of beam damage, although in this regard, efforts have been made to modify reactor designs,^34^ such as using a fluidized bed reactor^26,38,39^ and rotating the sample as a pellet.^34,35^

Our recent publication highlighted a new pathway to deactivation of H-SSZ-13 and H-SAPO-34 in MTH, where polyenes were shown to hinder diffusion of molecules in and out of the pores of the CHA topology during reaction.^40^ This is illustrated in Figure 1 where the formation of polyenes is coincident with a slowing of methanol consumption. We used a combination of spectroscopy and molecular simulations to identify intermediates and rationalize their behavior in the reaction. By the application of Kerr-gated Raman spectroscopy with an excitation wavelength of 400 nm, it was possible to remove sample background fluorescence and simultaneously take advantage of resonance enhancement effects to obtain strong signals from hydrocarbon pool species, which are known to absorb light around this same wavelength.^14,16−21^ This was a rare opportunity to use a visible-wavelength probe for Raman measurements on a system which is highly prone to fluorescence, enabled by the application of the Kerr-gated Raman spectrometer, which allowed fluorescence rejection on a picosecond time scale.^41^ For a comparison, in this paper, we show data collected during a repeated reaction of MTH over the H-SSZ-13 catalyst using a Raman probe of 267 nm instead, allowing a direct comparison of signals recorded with the UV Raman probe against the visible-wavelength probe (400 nm). The experimental conditions were kept constant as those in our previous publication,^40^ to offer a direct comparison of the hydrocarbon pool intermediates as are detectable by visible wavelength versus UV wavelength Raman probe. As in the previous experiments, during measurements, we used a raster to move the sample in the plane perpendicular to the beam, in a Lissajous pattern across a square of area 2 × 2 mm on the sample surface, avoiding measuring in one spot to minimize sample damage by the beam during the measurement.

**Figure 1:**
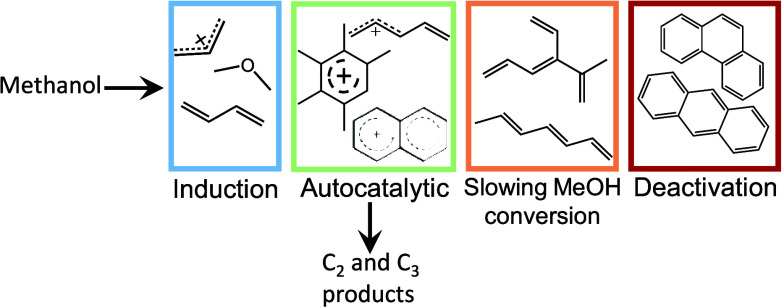
Schematic of the neutral and cationic molecular species observed in the general reaction mechanism of MTH.

*Operando* UV–vis experiments illustrate the importance of using different wavelengths for Raman measurements to capitalize on resonance enhancement effects. Figure S1 shows changing UV–vis spectra during MTH on H-SSZ-13 under temperature ramping conditions. In general, with increasing temperature and reaction time, the intensity of the absorbance bands increases, and more bands appear at longer wavelengths. This has been studied and discussed previously for MTH, specifically in H-SSZ-13 in multiple publications,^14−16,31,42^ and assignments have been made as discussed in the SI. Overall, the UV–vis data shows species that form during the reaction are distinguishable by their absorbance bands, and therefore, resonance Raman should be helpful to further distinguish these species by their molecular vibrations.

During the UV Raman experiment, changes were recorded upon methanol adsorption on H-SSZ-13 at 100 °C. Figure 2b shows Raman spectra recorded for the bare zeolite and the zeolite after methanol adsorption. The zeolite vibrations can be observed, with a strong symmetric stretch of Si–O–Si in the framework at 470 cm^–1^ and asymmetric stretch at 805 cm^–1^.^36^ The symmetric and asymmetric stretches of the CH_3_ group in methanol are detected at 2855 and 2955 cm^–1^. The C–O stretch in methanol is seen at 1003 cm^–1^ and its CH_3_ deformation at 1455 cm^–1^.^37,43^ These vibrations described so far are consistent with the experiment conducted with the 400 nm Raman probe (herein described as data collected during KG_400).^40^ In contrast to experiment KG_400, a strong band at 1620 cm^–1^ develops early on after methanol adsorption at 100 °C. Such a vibration could belong to the small hydrocarbons on the surface including possibly olefins as well as monoenylic and dienylic species, which are related with the start of a buildup of hydrocarbon pool species.^40^ Small olefins containing isolated C=C bonds absorb light in the UV region of the spectrum and would therefore be resonance enhanced by UV Raman; this would explain their detection by UV Raman and not by KG_400.^20^ A further vibration is recorded at 1180 cm^–1^ that may be associated with the collective C–H rocking in olefins, providing further support for the assignment. According to calculations by Lezcano-Gonzalez et al., this vibration is strong in small olefinic molecules, for example, butadiene.^40^

**Figure 2:**
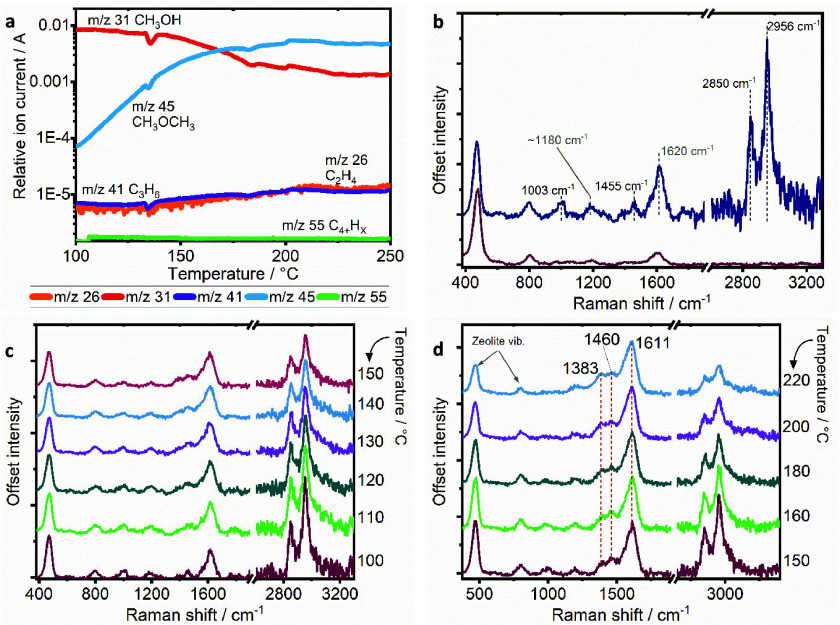
(a) *Operando* MS data collected in conjunction with UV Raman spectra during methanol conversion in the 100–250 °C range (*m*/*z* = mass-to-charge ratio). (b) Raman spectrum of calcined H-SSZ-13 catalyst activated at 600 °C in 20% oxygen (dark red line) and with methanol adsorbed at 100 °C (blue line). (c,d) Raman spectra collected in the temperature range from 100–150 and 150–220 °C during MTH reaction.

After methanol adsorption at 100 °C, a strong increase in the background was observed (as shown in Figure S2), related with the fluorescence of small amounts of hydrocarbons developing on the surface—this is in line with the formation of small olefins—although the background is low enough that it can be removed by a simple baseline subtraction to reveal the Raman spectrum shown in Figure 2b.

During the induction period of the reaction of this repeated experiment, we observed the same catalytic activity by MS as that published in ref (40), making this an ideal reference for comparison. Data in Figure 2a show MS data collected in the range from 100 to 250 °C, and the primary reaction is the formation of DME from the dehydration of methanol, while olefin signals remain stable and unchanging. This observation is in line with small hydrocarbons present on the surface that strongly interact with the zeolite, and remain on the surface, as would be typical for the induction period during the buildup of the hydrocarbon pool.^14,24,25^

As temperature is increased by 1 °C min^–1^, the methanol bands are gradually attenuated and replaced by those in the midrange of the spectrum, attributed to hydrocarbon intermediates. As shown in Figure 2c, by 150 °C, there is significant broadening of the C=C stretch and shift to lower frequencies (1607 cm^–1^) as the species formed encompass hydrocarbons that are more conjugated than those containing the isolated C=C bonds observed at 100 °C. In addition, two further bands develop, one at 1460 cm^–1^ where the ring stretching modes in cyclopentadienyl species are typically observed—which would be resonance enhanced at this wavelength,^36,44^ and another one at 1383 cm^–1^ in the region of CH_3_ and CH_2_ deformations, likely due to olefins.^40,45^ While the latter vibration would not be prone to resonance enhancement, not being directly a part of the chromophore, the C=C stretching in cyclopentadienyl species should be strongly enhanced, since such species exhibit an absorption maximum at 267 nm when neutral or 297 nm when protonated but with a band sufficiently broad as to cover the 267 nm probe laser wavelength.^46^ Our data collected and presented in Figure S1 do present growing absorbance in this region, although absorbance remains overall very low. We note here that while adsorbed methanol exhibits a CH_3_-deformation vibration near 1455 cm^–1^, the band does not decrease with the same trend as the CH stretches of methanol, confirming that the increasing intensity is not related to this methanol vibrational mode. As the temperature increases to 240 °C, the reaction remains in the induction period according to the MS data shown in Figure 4a. During this stage of reaction, the zeolite framework vibrations are seen to gradually drop as vibrations owing to carbonaceous intermediates on the surface grow, whereas the CH stretches of methanol diminish as it is consumed.

From a spectroscopic perspective, it is interesting to note that, in the KG_400 experiment, we were unable to detect strong signals from the induction period of the reaction. By probing with UV Raman, we are now able to measure strong signals from early hydrocarbon pool species that form prior to the autocatalytic period, including observing stronger signals from short-chain olefins and potentially cyclopentadienyl species, which went undetected at such low temperatures in the data presented in ref (40). For comparison, the data are replotted in Figure 3, overlaid with the UV Raman data. The dropping relative intensity of the zeolite framework vibrations is insignificant in KG_400, with the lesser degree of resonance enhancement, while during UV Raman, their relative intensity is more than halved as resonance enhancement promotes the signals of developing hydrocarbons that are building up in the reaction. The CH stretch modes of methanol are also further attenuated in this period by UV Raman as compared with KG_400.

**Figure 3:**
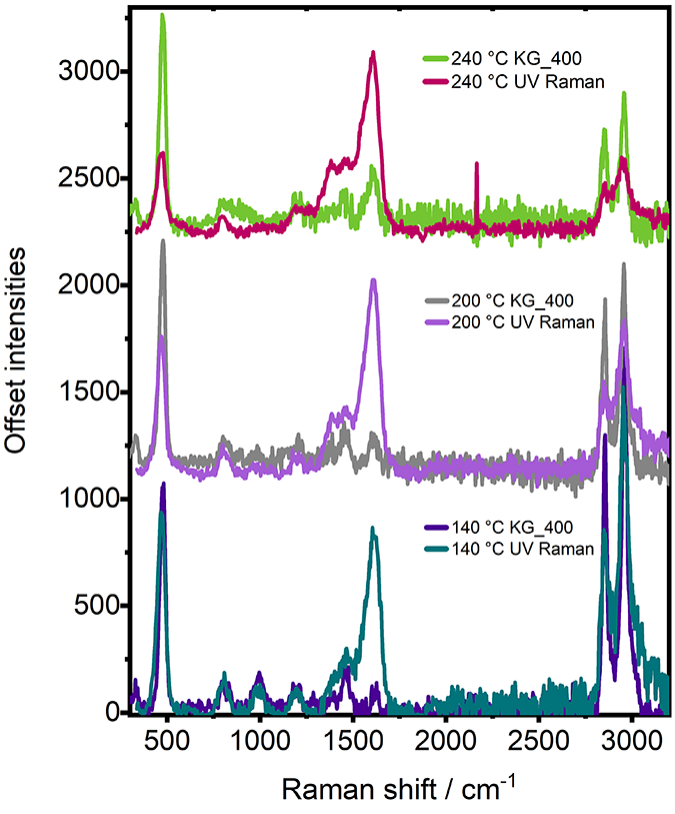
Overlaid Raman spectra of MTH at reaction temperatures of 140, 200, and 240 °C collected during KG_400 and UV Raman experiment.

Under these measurement conditions, we are able to observe the growing hydrocarbons involved prior to the autocatalytic period, where the signals were much weaker in KG_400. Prior literature has discussed precursors of the autocatalytic period by infrared spectroscopy,^47^ NMR,^25^ and UV–vis spectroscopy^14,46^ and indicate that small olefinic species are typically the first species detected on the zeolite surface. UV–vis spectroscopy has successfully detected cyclopentadienyl species in the reaction over H-ZSM-5 but as part of the autocatalytic regime, not the induction period.^44,46^ Here, the signals remain clear up to 240 °C, where the very weak CH stretches of methanol would suggest that this signal at 1460 cm^–1^ is a distinct species from methanol. Figure S3 shows how the relative intensities of the signals at 1006, 1460, and 2955 cm^–1^ change from 100 to 150 to 200 °C with respect to their starting intensities and illustrates the different behavior of the band at 1460 cm^–1^.

At 260 °C, by MS, a sudden increase in the signals at *m*/*z* 26, 41, and 55 indicates a sharp increase in the formation of olefins to a maximum point at 280 °C, as the methanol signal (*m*/*z* 31) drops, indicating higher conversion. Both changes occur as the catalyst enters its autocatalytic regime.

Raman spectra recorded from 240 to 260 °C—wherein increasing methanol conversion is observed—are shown in Figure 4b. At 250 °C, a strong C=C stretch at 1605 cm^–1^ dominates the spectrum, with the broad shoulder at 1380 cm^–1^ still relating to the deformations of CH_2_ and CH_3_ groups.^45^ Other species with vibrations in this region would be naphthalenic species, which have been shown to be active to some degree in hydrocarbon pool chemistry in the CHA topology, although they would not be expected at such early stages, such as the induction period. As the temperature is increased to 260 and 270 °C, the vibrations of the zeolite framework further weaken, with the C=C stretch becoming relatively much stronger. In the low frequency region of Figure 4c, note the weak band at 550 cm^–1^, which theoretical calculations predicted to be caused by the C–C–C bending modes of alkyl groups on aromatics or alkylated dienes.^40^ It can be difficult to distinguish monocyclic aromatic hydrocarbons from branched olefins, since they share many common vibrational modes; however, during KG_400, the difference became clearer because there was a huge increase in resonance-enhanced signals once the branched, aromatic hydrocarbons had formed at 270 °C.^40^ In this experiment, the formation of monocyclic aromatic hydrocarbons, which are well-established to be the main driving species in the reaction,^14,15,27−29^ are more difficult to identify. Rather, we can identify only more general branched carbenium ions, by the alkyl group signal at 550 cm^–1^.^40^

**Figure 4:**
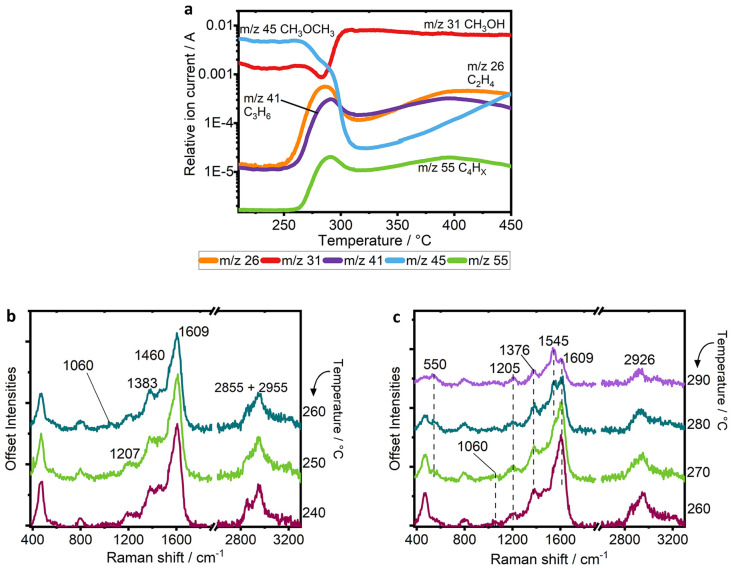
(a) MS data collected from 230 to 450 °C during the reaction of MTH on H-SSZ-13 under temperature ramping, (b) UV Raman spectra collected from 240–260 °C, and (c) UV Raman spectra collected from 260–290 °C.

At 280 °C, a new signal emerges in the C=C stretch region at 1545 cm^–1^, in close frequency to a band observed during KG_400 at 1551 cm^–1^ corresponding to long-chain polyenes.^40,48^ The fact that we are able to more clearly observe this band during the UV Raman experiment might be related with the lesser resonance enhancement of methylated benzenium ions, or perhaps the wavelength of the 267 nm excitation is closer to the absorption maximum of these species so that when they are in low concentration they are more easily detectable at 280 °C. By 290 °C, the vibration at 1545 cm^–1^ becomes stronger than that at 1609 cm^–1^ indicating that the relative concentration of polyenes has increased. Polyenes were first observed by Chua and Stair by UV Raman, who first postulated an assignment to polyenes,^48^ before Lezcano-Gonzalez et al. were finally able to confirm the assignment and linked the species with a role in catalyst deactivation by combining *operando* studies and advanced molecular simulations.^40^ Polyenes were found to contribute initially to catalyst deactivation through blocking windows to the active chabazite cages, preventing entry of methanol or diffusion of the small olefin products out of the zeolite^40^ and explaining the drop in methanol conversion that we observe by MS. It is important to note again that the drop in methanol consumption occurred prior to the detection of any polycyclic aromatic hydrocarbons, which were generally thought to be responsible for catalyst deactivation.^22,30,36^

Polyenes also play a secondary role in catalyst deactivation whereby as the reaction proceeds, polyenes can cyclize to form polyaromatic hydrocarbons,^40^ which have long since been understood to form inside zeolites of the chabazite topology in this reaction.^14,30^ This is shown again beyond 300 °C, as the relative intensity of the 1545 cm^–1^ band drops, and a strong band at 1391 cm^–1^ grows significantly, with a new, strong band at 1615 cm^–1^, which together indicate the formation of polycyclic aromatic hydrocarbons, characterized by their ring-breathing mode and C=C stretch in the ring, respectively.^11,40,48^

Figure 5b shows Raman spectra collected after the catalyst enters deactivation with decreasing methanol conversion. During this time, the Raman bands become more typical of those observed during the analysis of coke or bulk carbon, as the trapped species in the zeolite pores grow and become bulkier. The main spectral features maintain strong signals at 1620 and 1375 cm^–1^, whereas a shoulder present at higher temperatures at 1445 cm^–1^ is in agreement with the formation of polyaromatics, assigned to polyaromatic hydrocarbons that contain a bent structure such as fluorene or phenanthrene.^9^ Coke deposits in MTH using zeolite catalysts with the CHA topology are discussed in the literature and identified as polyaromatic hydrocarbons that fill the pore structure internally. Such species include methylated naphthalene, pyrene and phenanthrene.^30,31^

**Figure 5:**
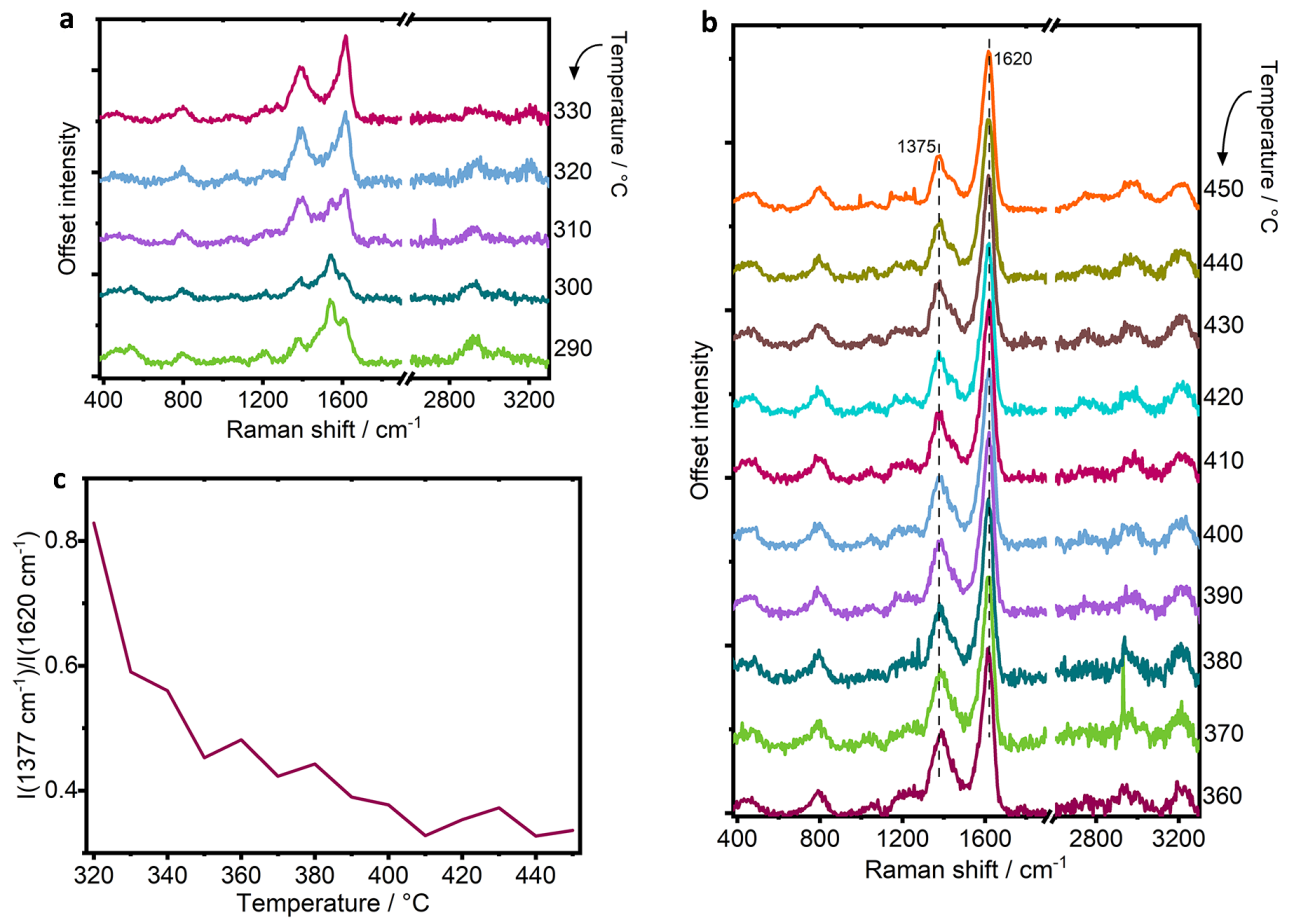
Raman spectra after the onset of deactivation is reached (a) from 290 to 330 °C and (b) from 360 to 450 °C, and (c) plot of relative intensity of vibrations 1377 vs 1620 cm^–1^ toward the higher temperatures at the end of the experiment.

At high frequency, three bands develop at 2755, 2965, and 3215 cm^–1^, which are second-order Raman bands of the carbon deposits. The band at 2755 cm^–1^ is an overtone of the ring-breathing mode at 1370 cm^–1^, that at 2965 cm^–1^ is a combination band of the ring-breathing mode and symmetric C=C stretch at 1615 cm^–1^, while that at 3215 cm^–1^ is an overtone of the symmetric C=C stretch.^10^ These second order vibrations are commonly observed in graphitic-type carbonaceous species.^10^

In reference to experiment KG_400, with increasing temperature, the ring-breathing mode of polyaromatic hydrocarbons (ca. 1375 cm^–1^) remained very strong and the evolution of polyaromatics is continuously observed by a strong low frequency vibration at 630 cm^–1^.^40^ The spectrum for comparison is plotted in Figure 6a, collected at the end of the temperature ramp at 450 °C. By UV Raman, the strong signal at 630 cm^–1^ is circumvented, and the ring-breathing mode of polyaromatics is weakened. These differences come again with differences in resonance enhancement, and although the polyaromatic hydrocarbons still exhibit strong absorption bands in the UV region, they come to serve as an example of how resonance enhancement is specific to the chromophore as opposed to the entire molecule.^7^ With a UV Raman probe, we see significant changes in the relative intensity of vibrations of the general C=C stretch and ring-breathing mode and an absence of the low frequency vibrations of polyaromatics as we approach 450 °C. Figure 5c shows a plot of the relative intensities of the signals at 1377 and 1620 cm^–1^ with increasing temperature to illustrate the change. The changes correlate with comments by Signorile et al., who noted a decrease in the intensity of the band in the 1320–1380 cm^–1^ region of polyaromatic hydrocarbons by moving to a shorter wavelength Raman probe.^11^

**Figure 6:**
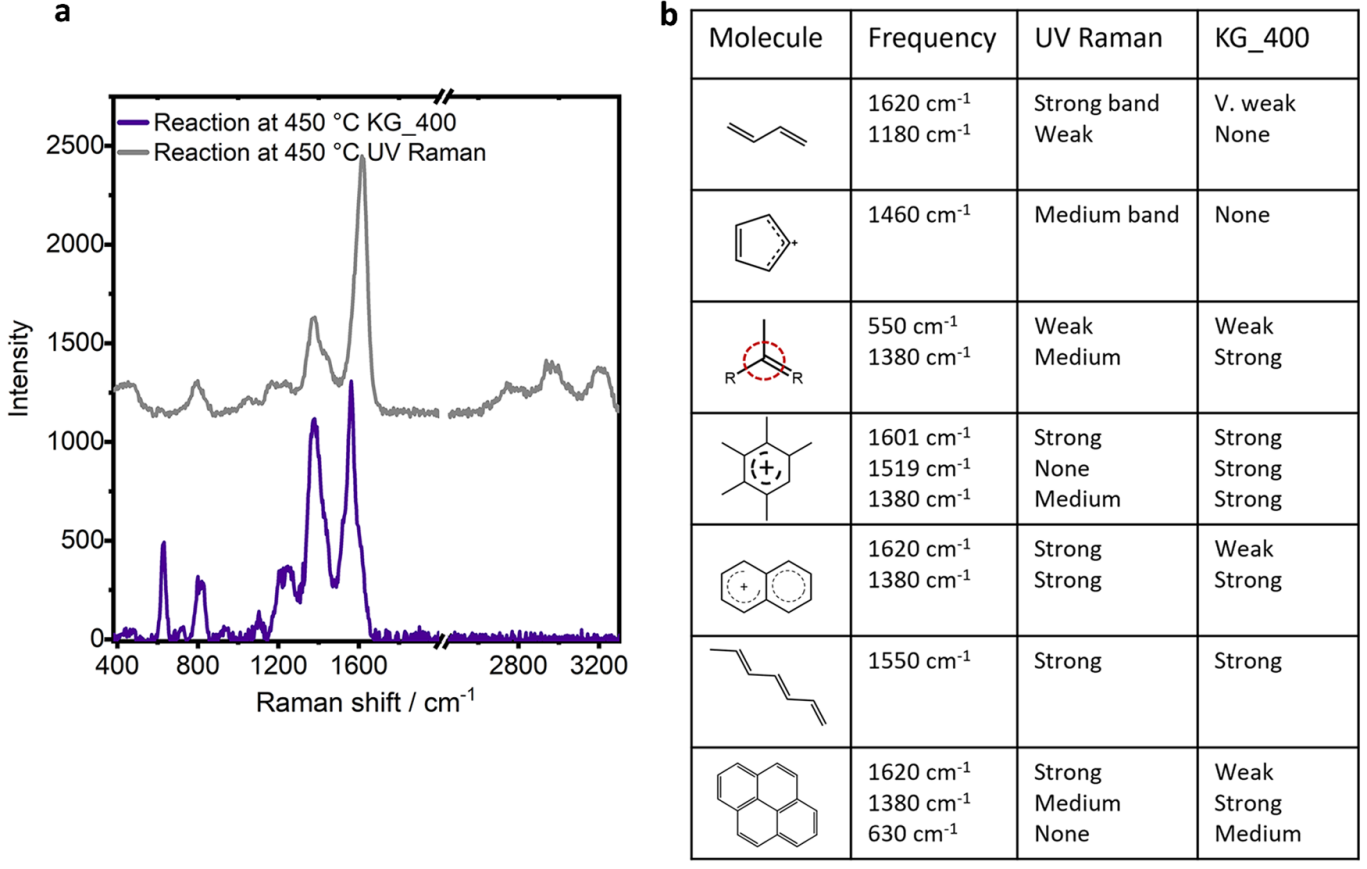
(a) Comparison of Raman spectra collected by UV wavelength probe with that collected with 400 nm probe by Kerr-gated Raman, during the reaction of MTH on H-SSZ-13, and (b) a summarizing table of species or functional groups observed during UV Raman and/or KG_400.

In general, when Raman is used to describe bulk carbon, the ratio of the D band (1320–1380 cm^–1^) to the G band (1500–1630 cm^–1^) can be used to describe the degree of graphitic or amorphous nature of the carbon.^10,49,50^ The D band in general arises from the breathing modes of aromatic rings, while the G band arises from the bond stretching of pairs of sp^2^ hybridized carbon pairs and can be attributed to both aromatic and olefinic species.^10,49,50^ The bands will vary in intensity and position as the type of carbon probed changes. In general, at any fixed wavelength, the ratio of the intensities of the D to G band should decrease with increasing graphitization, i.e., growing size of the polyaromatic hydrocarbon structure.^10^ This is illustrated by Figure 5c and confirms that, with increasing temperature, we see polyaromatic hydrocarbons whose cluster sizes grow.

The relative intensity of the bands may also change when varying the excitation energy, and/or the frequency of the G band may change, offering deeper insight.^10,11^ Generally, by moving to a shorter wavelength, with probing polyaromatic hydrocarbons, the intensity ratio of the D to G band is decreased. This effect is shown by comparing spectra of the catalyst at the end of the reaction as probed by UV Raman and visible Raman, where the data is given in Figure 6a, and shows a much decreased ratio of D to G band by UV Raman. This gives us additional confidence that with increasing temperature we indeed see polyaromatic hydrocarbons with growing cluster size.

When comparing the Raman results collected using the 267 or 400 nm wavelengths as a probe into MTH in H-SSZ-13, many similar species were detected, including branched carbenium ions, polyenes, and polycyclic aromatic hydrocarbons. Stronger vibrations were observed at lower temperatures of reaction during the induction period by 267 nm excitation, which were not observed at 400 nm, namely, short-chain olefins and possibly cyclopentadienyl cations. The 400 nm probe was more insightful in determining the point of monocyclic aromatic hydrocarbon formation, as this is the first of the species absorbing at 400 nm. Polyaromatic hydrocarbons could be distinguished by their low frequency vibration at 630 cm^–1^ with a 400 nm probe that was absent by UV Raman. Figure 6b summarizes the vibrations that were identifiable with each wavelength. In describing carbonaceous deposits, the application of both UV Raman and visible-wavelength Raman can give a more complete description of the nature of such deposits, as is highlighted by the comparison of deactivated catalysts. Although for such measurements with visible-wavelength probe, a gating system is required with a very fast fluorescence rejection rate to circumvent fluorescence, such as a Kerr-gated spectrometer. The results overall highlight the importance of using different wavelengths to exploit resonance enhancement, giving a comprehensive view of intermediates in such complex mechanisms.

## Methods

H-SSZ-13 was synthesized according to ref (51), but under static conditions, and was the same batch of material used in ref (40). The zeolite was calcined under static conditions in air (1 °C min^–1^ to 120 °C and hold for 2.5 h, 2.2 °C min^–1^ to 350 °C and hold for 3 h, then 0.8 °C min^–1^ at 580 °C and hold for 3 h). The resulting material was characterized by powder X-ray diffraction, scanning electron microscopy, N_2_ sorption, inductively coupled plasma optical emission spectrometry (ICP-OES), and ^27^Al MAS NMR.^52^

Catalytic reactions were carried out in a commercial Linkam CCR1000 stage. 50 mg of catalyst was loaded into the sample holder, on top of the ceramic fiber filter, and gently pressed. After the catalyst pretreatment in 20% O_2_/He at 550 °C for 1 h, the catalyst was flushed with He and cooled to 100 °C. A He stream was maintained at 30 mL min^–1^, and then, methanol was injected into the He stream at 1.7 μL min^–1^ continuously by means of a syringe pump. The temperature was increased linearly from 100 to 450 °C at 1 °C min^–1^. Catalytic activity was recorded by online mass spectrometry (MS) using a Pfeiffer Omnistar mass spectrometer; mass-to-charge (*m*/*z*) values reported in this work are 26, 31, 41, 45, and 55, to correspond to major mass fragments in ethylene, methanol, propylene, dimethyl ether, and butanes/butenes, respectively.

UV–vis data was collected on a modular setup from Ocean Optics and using the CCR1000 Linkam Cell as described above. The UV–vis setup comprises a Flame-S-XR1-ES Ocean Optics spectrometer with a 100 μm slit, DH-2000-S-SUV-TTL light source, and QR400- 7-SR-BX reflection probe (fiber optic probe). The probe was held to the quartz window such that the excitation light hit the sample at a 45° angle, collecting back the diffusely reflected light at that same angle. BaSO_4_ was loaded into the CCR1000 instrument to collect a background for the measurements.

The new UV Raman data reported in this paper were acquired on the same custom-built spectrometer with some modifications as was used in ref (40) at the ULTRA facility, RAL UK,^41^ using the third harmonic of the picosecond Ti-Sapphire laser to generate the 267 nm wavelength probe. The laser was operated at a 10 kHz repetition rate, producing picosecond pulses (2 ps pulse length) at an 800 nm fundamental wavelength. The 267 nm probe beam power at the sample was attenuated to 1 mW, which corresponds to a 100 nJ pulse energy. In contrast to what was reported in ref (40), for the present UV Raman, the experiment was performed with the Kerr gate removed from the setup, and all the glass signal collection optics and visible-range mirrors were replaced with the fused silica lenses and UV mirrors, respectively. The collected signal beam was dispersed with a diffraction-grating spectrograph and detected with a UV-enhanced CCD. The data was accumulated for a total of 60 s. Raman measurements were taken at 10 °C intervals during the MTH reaction. The Linkam stage was attached to a raster system to move the sample in the plane perpendicular to the beam to avoid laser damage during the measurements. The laser spot size at sample was approximately 150 μm, and it was rastered across 2 × 2 mm area following a Lissajous pattern. This way, the area irradiated by the laser was expanded significantly, and the sample was irradiated in a near-random pattern, and the effectiveness of the raster in preventing sample damage is illustrated in Figure S4.

## Supporting Information Available

Additional information regarding absorbance bands assigned in literature for species involved in the MTH reaction and our UV–vis data collected during MTH on H-SSZ-13, Raman data collected before and after methanol adsorption and comparison of the changing relative intensities of bands relating to methanol, and finally, figures that show the benefit of using the raster in avoiding damage by the laser to the sample (PDF)
Transparent Peer Review report available (PDF)
