# Supplementary material for: Methanol-to-Olefins Studied by UV Raman Spectroscopy as Compared to Visible Wavelength: Capitalization on Resonance Enhancement
Source: J Phys Chem Lett. 2024 Jun 25;15(26):6826–34. doi: 10.1021/acs.jpclett.4c00865 (PMC11229064; doi:10.1021/acs.jpclett.4c00865)
Supplement: Supplementary file 1 — jz4c00865_si_001.pdf [file jz4c00865_si_001.pdf]

Supplementary information for

Methanol-to-Olefins Studied by UV Raman Spectroscopy as Compared to Visible Wavelength; Capitalisation on Resonance Enhancement

Emma Campbell [1,2], Michael Towrie[4], Igor Sazanovich[4], Michael J. Watson[5], Ines Lezcano-Gonzalez[2,3], Andrew M. Beale[2,3]

[1]Cardiff Catalysis Institute School of Chemistry, Cardiff University, Cardiff, CF10 3AT, UK

[2]*Department of Chemistry, University College London, 20 Gordon Street, London, WC1H 0AJ, UK*

[3]*Research Complex at Harwell (RCaH), Harwell, Didcot, Oxfordshire, OX11 0FA, UK*

[4]*Central Laser Facility, Research Complex at Harwell, Rutherford Appleton Laboratories, Harwell Campus, OX11 0QX, UK*

[5]*Johnson Matthey Technology Centre, P O Box 1, Belasis Avenue, Billingham TS23 1LB*

Table S1 – a summary of the absorbance maxima for lowest energy transitions observed for UV-Vis for a range of compounds with ranging conjugation, or neutral or protonated. Summarised from literature.[1–7]

Level of conjugation	Example species	Wavelength of HOMO→LUMO transition / nm
1 double bond	Alkene (eg propene)	<190 nm
2 conjugated double bonds	Diene (eg butadiene)	220 – 245 nm
3 conjugated double bonds	Triene, monocyclic aromatic hydrocarbon	250 – 290 nm
5 conjugated double bonds	Decapentaene, naphthalene	280 – 350 nm
1 double bond + positive charge	Dienylic cation (eg protonated butadiene)	280 – 330 nm
2 double bonds + positive charge	Methylated benzenium ions	360 – 390 nm
3 double bonds + positive charge	Methylated naphthalene cations	430 – 470 nm
5 double bonds + positive charge	Cations of polyaromatic hydrocarbons eg phenanthrene	>540 nm

Figure S1 UV-Vis data acquired during MTH on H-SSZ-13 from 100 to 450 °C, (inset shows 140 to 240 °C due to the much lower absorbance).

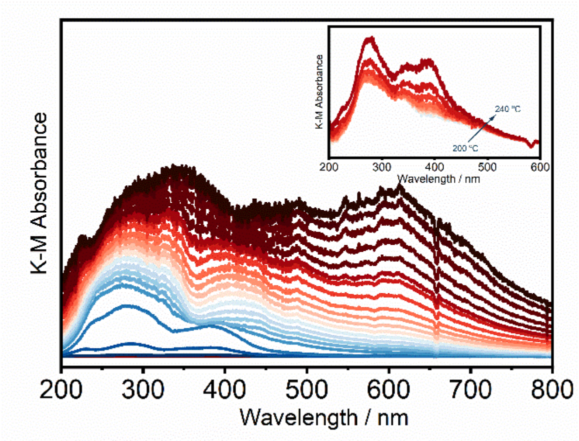

Initially, very low absorbance is recorded with a maximum at 275 nm that corresponds to the formation of monoenylic carbocations or cyclopentadienyl carbocations;[1,8] in the spectrum collected at 200 °C, two further bands with maxima at 345 and 390 nm are recorded which have been assigned to the presence of dienyl carbocations or low-methylated benzenium ions, and highly-methylated benzenium ions, as the hydrocarbon pool starts to build.[1,2,9] From 200 to 240 °C (spectra shown in the inset of **Error! Reference source not found.**) the relative intensity of these bands change, those at 345 and 390 nm becoming stronger which would correspond to the build-up of methylated benzenium ions as the reaction proceeds and temperature increases.[1,10,11] At 260 and 270 °C, there are drastic increases in intensity across the spectrum. At 290 °C, absorbance at longer wavelengths becomes clear, and a new maximum at 415 nm signals the formation of methylated naphthalenic cations, and at 310 °C onwards absorbance further into the visible indicates the development of protonated polyaromatic hydrocarbons (possibly including anthracene or phenanthrene) which absorb at 590 – 615 nm.[1,10,11]

Figure S2 – Raw spectra collected without background subtraction before and after methanol adsorption

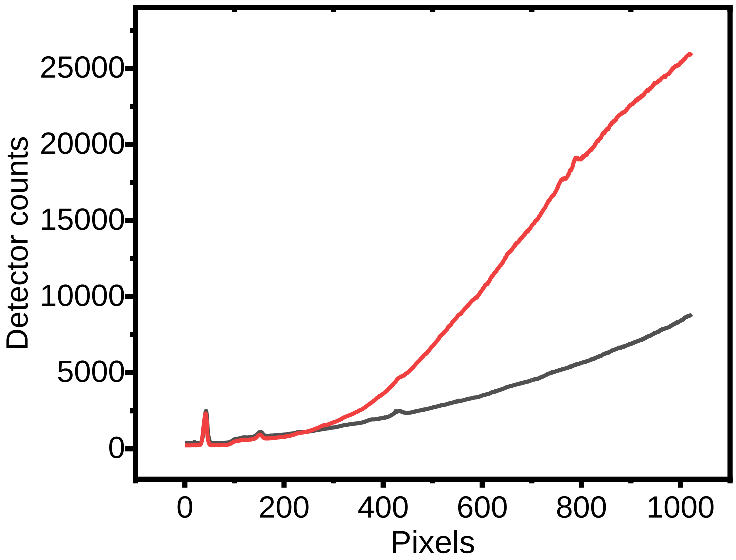

Figure S3 – Relative intensity of signals at 1006, 2955 and 1460 cm^{-1} at 100, 150 and 200 °C, to illustrate the different rate of change of the band at 1460 cm^{-1} to other methanol vibrational modes.

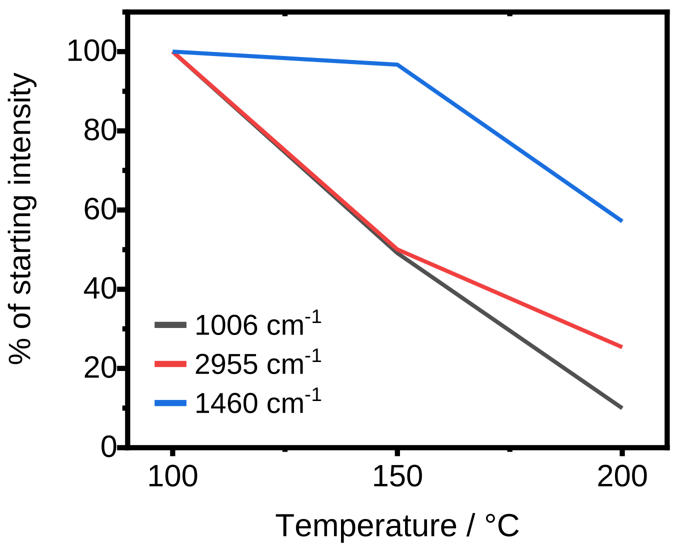

Figure S4 – Spectra collected using the Kerr-gated Raman spectrometer (400 nm Raman probe) to measure a spectrum on toluene adsorbed on silica, showing the effect of the rastering system in protecting the sample from beam damage. Without the rastering system the spectrum can only be recorded for the first 20s of accumulation before the toluene degrades.

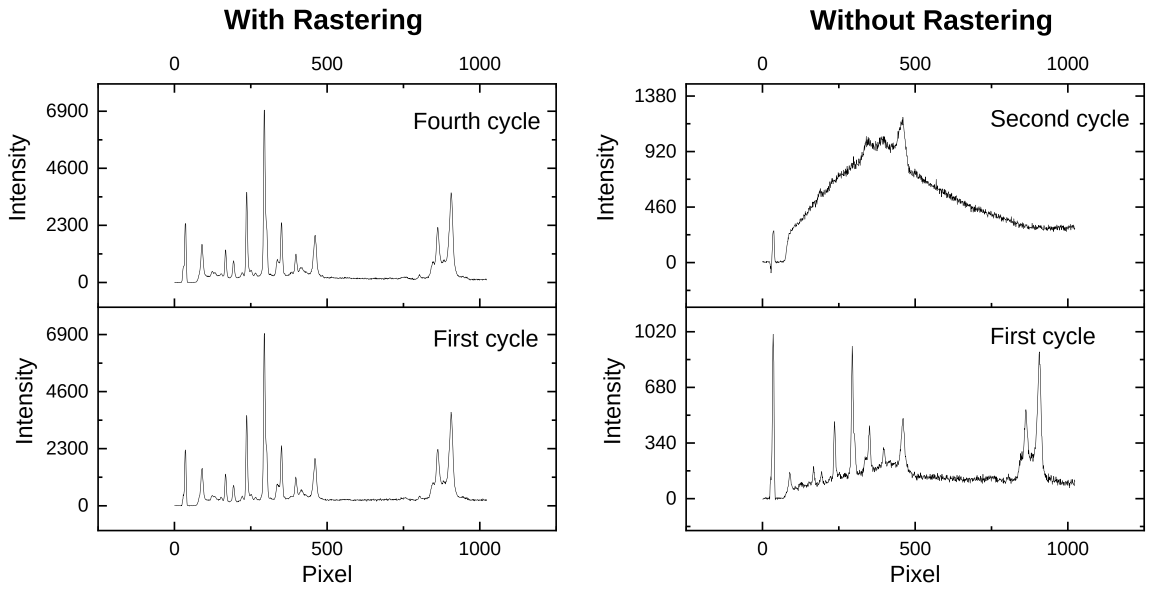

Data availability statement: Original data from the spectra and traces shown in the manuscript and the supplementary information can be found via the following web address: https://doi.org/10.5522/04/25991689.v1

References

(1) Borodina, E.; Meirer, F.; Lezcano-González, I.; Mokhtar, M.; Asiri, A. M.; Al-Thabaiti, S. A.; Basahel, S. N.; Ruiz-Martinez, J.; Weckhuysen, B. M. Influence of the Reaction Temperature on the Nature of the Active and Deactivating Species during Methanol to Olefins Conversion over H-SSZ-13. *ACS Catal.* **2015**, *5* (2), 992–1003. https://doi.org/10.1021/cs501345g.

(2) Goetze, J.; Meirer, F.; Yarulina, I.; Gascon, J.; Kapteijn, F.; Ruiz-Martínez, J.; Weckhuysen, B. M. Insights into the Activity and Deactivation of the Methanol-to-Olefins Process over Different Small-Pore Zeolites As Studied with Operando UV–Vis Spectroscopy. *ACS Catal.* **2017**, *7* (6), 4033–4046. https://doi.org/10.1021/acscatal.6b03677.

(3) Geobaldo, F.; Spoto, G.; Bordiga, S.; Lamberti, C.; Zecchina, A. Propene Oligomerization on H-Mordenite: Hydrogen-Bondinginteraction, Chain Initiation, Propagation and Hydrogen Transferstudied by Temperature-Programmed FTIR and UV–VISspectroscopies. *J. Chem. Soc. Faraday Trans.* **1997**, *93* (6), 1243–1249. https://doi.org/10.1039/A607052C.

(4) Borodina, E.; Sharbini Harun Kamaluddin, H.; Meirer, F.; Mokhtar, M.; Asiri, A. M.; Al-Thabaiti, S. A.; Basahel, S. N.; Ruiz-Martinez, J.; Weckhuysen, B. M. Influence of the Reaction Temperature on the Nature of the Active and Deactivating Species During Methanol-to-Olefins Conversion over H-SAPO-34. *ACS Catal.* **2017**, *7* (8), 5268–5281. https://doi.org/10.1021/acscatal.7b01497.

(5) Qian, Q.; Vogt, C.; Mokhtar, M.; Asiri, A. M.; Al-Thabaiti, S. A.; Basahel, S. N.; Ruiz-Martínez, J.; Weckhuysen, B. M. Combined Operando UV/Vis/IR Spectroscopy Reveals the Role of Methoxy and Aromatic Species during the Methanol-to-Olefins Reaction over H-SAPO-34. *ChemCatChem* **2014**, *6* (12), 3396–3408. https://doi.org/10.1002/cctc.201402714.

(6) Garratt, P. J. *UV-Vis Atlas of Organic Compounds*, 2nd Ed.; Heinz-Helmut Parkampus: Weinheim, 1992; Vol. 5.

(7) Yang, S.; N. Kondo, J.; Domen, K. Formation of Stable Alkenyl Carbenium Ions in High Yield by Adsorption of 1-Methylcyclopentene on Zeolite Y at Low Temperature. *Chem. Commun.* **2001**, *0* (19), 2008–2009. https://doi.org/10.1039/B106450A.

(8) Hernandez, E. D.; Jentoft, F. C. Spectroscopic Signatures Reveal Cyclopentenyl Cation Contributions in Methanol-to-Olefins Catalysis. *ACS Catal.* **2020**, *10* (10), 5764–5782. https://doi.org/10.1021/acscatal.0c00721.

(9) Bjørgen, M.; Bonino, F.; Kolboe, S.; Lillerud, K.-P.; Zecchina, A.; Bordiga, S. Spectroscopic Evidence for a Persistent Benzenium Cation in Zeolite H-Beta. *J. Am. Chem. Soc.* **2003**, *125* (51), 15863–15868. https://doi.org/10.1021/ja037073d.

(10) Hemelsoet, K.; Qian, Q.; De Meyer, T.; De Wispelaere, K.; De Sterck, B.; Weckhuysen, B. M.; Waroquier, M.; Van Speybroeck, V. Identification of Intermediates in Zeolite-Catalyzed Reactions by In Situ UV/Vis Microspectroscopy and a Complementary Set of Molecular Simulations. *Chem. – Eur. J.* **2013**, *19* (49), 16595–16606. https://doi.org/10.1002/chem.201301965.

(11) Van Speybroeck, V.; Hemelsoet, K.; De Wispelaere, K.; Qian, Q.; Van der Mynsbrugge, J.; De Sterck, B.; Weckhuysen, B. M.; Waroquier, M. Mechanistic Studies on Chabazite-Type Methanol-to-Olefin Catalysts: Insights from Time-Resolved UV/Vis Microspectroscopy Combined with Theoretical Simulations. *ChemCatChem* **2013**, *5* (1), 173–184. https://doi.org/10.1002/cctc.201200580.
