# Supplementary material for: Methanol-to-Olefins Studied by UV Raman Spectroscopy as Compared to Visible Wavelength: Capitalization on Resonance Enhancement
Source: J Phys Chem Lett. 2024 Jun 25;15(26):6826–34. doi: 10.1021/acs.jpclett.4c00865 (PMC11229064; doi:10.1021/acs.jpclett.4c00865)
Supplement: Supplementary file 2 — jz4c00865_si_002.pdf [file jz4c00865_si_002.pdf]

jz-2024-008656.R1

Name: Peer Review Information for "Methanol-to-Olefins Studied by UV Raman Spectroscopy as Compared to Visible Wavelength; Capitalisation on Resonance Enhancement"

First Round of Reviewer Comments

Reviewer: 1

Comments to the Author

In this work, the authors systemically investigated the reaction mechanism of methanol conversion using resonance Raman spectroscopy. The Raman bands associated with different intermediates formed in the induction stage, steady stage and deactivation stage of MTH were identified. It was found that the UV excitation enhances the peaks of light olefins and cyclic hydrocarbon species (e.g. cyclopentadienyl cations), whereas for visible excitation, it is more suitable for description of bulky aromatic species. I don't find obvious issue with this manuscript, and it can be accepted after some minor improvements.

1. I suggest that the authors expand the experimental section, especially catalyst preparation, characterization and catalytic testing.

2. Some Figures are not clear enough. Please improve the quality of the Figures in the whole work.

3. The authors are expected to carefully review the manuscript content, including spelling, formatting, and references.

Reviewer: 2

Comments to the Author

The manuscript by Campbell et al addresses the use of (resonance) UV Raman spectroscopy in the context of the methanol to olefin conversion over chabazite SSZ-13. In this temperature-dependent study Raman spectroscopic changes are discussed together with simultaneously recorded MS data.

While the study is interesting and conclusions are mostly supported by the data (see below) it does not reflect the novelty and immediate significance required for J Phys Chem Lett for the following reasons: The use of UV Raman spectroscopy was demonstrated previously for the same reaction on a similar small-pore chabazite zeolite (SAPO-34). In fact, there is an article by Signorile et al

(Catal Today 2019) not cited in the manuscript, which is devoted to the methodical aspects of using UV Raman spectroscopy for methanol to olefin conversion over SAPO-34. While there may be some interesting aspects related to the use of SSZ-13 not reported previously I consider the overall novelty of the current manuscript in terms of the application of UV Raman spectroscopy as limited.

In their manuscript the authors stress the importance of resonance conditions. To this end, however, no experimental support (that is UV-Vis spectra) is provided. In principle, a rigorous discussion would require the recordance of operando UV-Vis spectra to reflect the current state of the catalyst. Unfortunately, detailed catalytic (conversion, selectivity) and Raman spectroscopic (objective, laser intensity, spectral resolution) conditions are not reported in the manuscript. In particular, as no fluidized bed is used, detailed experimental conditions for the use of UV Raman spectroscopy need to be provided. To the best of my knowledge, the manuscript does not fit the J Phys Chem Letter format.

The manuscript may be considered for publication in J Phys Chem C subject to further review. When preparing a revised version, the authors should update the cited literature regarding regular as well as review articles containing UV-Raman spectroscopy.

Author's Response to Peer Review Comments:

DR ANDREW M. BEALE

26 May 2024

Dear Editor,

I am writing to resubmit our manuscript entitled "Methanol-to-Olefins studied by UV Raman spectroscopy as compared to visible wavelength; capitalisation on resonance enhancement" for consideration for publication in the prestigious ACS Journal of Physical Chemistry Letters.

Detailed below are our responses to the referee reports for jz-2024-008656. We thank the referees for providing us with their comments and suggestions that have allowed us to improve the content and clarity of the manuscript. We elaborate on the individual points raised below:

Response to reviewer #1

In this work, the authors systemically investigated the reaction mechanism of methanol conversion using resonance Raman spectroscopy. The Raman bands associated with different intermediates formed in the induction stage, steady stage and deactivation stage of MTH were identified. It was found that the UV excitation enhances the peaks of light olefins and cyclic hydrocarbon species (e.g. cyclopentadienyl cations), whereas for visible excitation, it is more suitable for description of bulky aromatic species. I don't find obvious issue with this manuscript, and it can be accepted after some minor improvements.

Comment 1. I suggest that the authors expand the experimental section, especially catalyst preparation, characterization and catalytic testing.

Response. We would like to thank the reviewer for this suggestion. We have now expanded the experimental section accordingly, which reads as follows:
"H-SSZ-13 was synthesised according to reference 51, but under static conditions, and was the same batch of material used in reference 40. The zeolite was then calcined under static conditions in air (1 °C min-1 to 120 °C and hold 2.5 h, 2.2 °C min-1 to 350 °C and hold 3 h, then 0.8 °C min-1 at 580 °C and hold for 3 h). The material was characterised by powder Xray diffraction, scanning electron microscopy, N2 sorption, inductively coupled plasma optical emission spectrometry (ICP-OES), and 27Al MAS NMR.[52]

Catalytic reactions were carried out in a commercial Linkam CCR1000 stage. 50 mg of catalyst was loaded into the sample holder, on top of the ceramic fiber filter, and gently pressed. After the catalyst pre treatment in 20 % O2/He at 550 °C for 1 h, the catalyst was flushed with He and cooled to 100 °C. He stream was maintained at 30 ml min^{-1}, then methanol was injected into the He stream at 1.7 µL min^{-1} continuously by means of a syringe pump. The temperature was increased linearly from 100 to 450 °C at 1 °C min^{-1}. Catalytic activity was recorded by on-line mass spectrometry (MS) using a Pfeiffer Omnistar mass spectrometer, mass-to-charge (m/z) values reported in this work are 26, 31, 41, 45, and 55, to correspond to major mass fragments in ethylene, methanol, propylene, dimethyl ether and butanes/butenes, respectively. UV-Vis data was collected on a modular setup from Ocean Optics and using the CCR1000

Linkam Cell as described above. The UV-Vis setup comprises a Flame-S-XR1-ES Ocean

Optics spectrometer with a 100 µm slit, DH-2000-S-SUV-TTL light source and QR400- 7-SRBX reflection probe (fibre optic probe). The probe was held to the quartz window such that the excitation light hit the sample at a 45 ° angle, collecting back the diffusely reflected light at that same angle. BaSO4 was loaded into the CCR1000 to collect a background for the measurements.

The new UV Raman data reported in this paper was acquired on the same custom-built spectrometer with some modifications as was used in ref 40 at the ULTRA facility, RAL UK,[41] using the 3rd harmonic of the picosecond Ti-Sapphire laser to generate the 267 nm wavelength probe. The laser was operated at 10 kHz repetition rate, producing picosecond pulses (2 ps pulse length) at 800 nm fundamental wavelength. The 267 nm probe beam power at the sample was attenuated to 1 mW which corresponds to 100 nJ pulse energy. In contrast to what was reported in 40, for the

present UV Raman the experiment was performed with the Kerr gate removed from the setup, and all the glass signal collection optics and visible-range mirrors replaced with the fused silica lenses and UV mirrors respectively. The collected signal beam was dispersed with a diffraction-grating spectrograph and detected with a UV-enhanced CCD. The data was accumulated for a total of 60 s. Raman measurements were taken at 10 °C intervals during the MTH reaction. The Linkam stage was attached to a raster stage so that to move the sample in the plane perpendicular to the beam to avoid laser damage during the measurements. The laser spot size at sample was approx. 150 µm and it was rastered across 2 x 2 mm area following Lissajous pattern. This way the area irradiated by the laser was expanded significantly and the sample was irradiated in a near random pattern, and the effectiveness of the raster in preventing sample damage is illustrated in Figure S4."

Comment 2. Some Figures are not clear enough. Please improve the quality of the Figures in the whole work.

Response. Thank you for the suggestion. The figures in the manuscript have been modified so as to improve the quality.

Comment 3. The authors are expected to carefully review the manuscript content, including spelling, formatting, and references.

Response. We thank reviewer 1 for pointing this out, we have now reviewed the manuscript spelling, formatting and references.

Response to reviewer #2

The manuscript by Campbell et al addresses the use of (resonance) UV Raman spectroscopy in the context of the methanol to olefin conversion over chabazite SSZ-13. In this temperaturedependent study Raman spectroscopic changes are discussed together with simultaneously recorded MS data.

While the study is interesting and conclusions are mostly supported by the data (see below) it does not reflect the novelty and immediate significance required for J Phys Chem Lett for the following reasons:

Comment 1. The use of UV Raman spectroscopy was demonstrated previously for the same reaction on a similar small-pore chabazite zeolite (SAPO-34). In fact, there is an article by Signorile et al (Catal Today 2019) not cited in the manuscript, which is devoted to the methodical aspects of using UV Raman spectroscopy for methanol to olefin conversion over SAPO-34. While there may be some interesting aspects related to the use of SSZ-13 not reported previously I consider the overall novelty of the current manuscript in terms of the application of UV Raman spectroscopy as limited.

Response. We consider that the unique combination of UV Raman and visible wavelength Kerr-Gated Raman presented in this work provides novel insight into the formation and evolution of hydrocarbon pool species, especially on the effect of the Raman excitation on the type of species detected. This is particularly relevant within the context of our previous work (Lezcano et al., Nat. Mater. 2020), in which the combination with theoretical methods allowed us to unambiguously assign the full Raman spectra when using a visible excitation. Nevertheless, the low Raman intensities observed at the early stages of the reaction prevented us to draw definitive conclusions in this period, namely the identification of precursors of the aromatic cycle. By combining UV Raman and visible wavelength Kerr-Gated Raman, we have now been able to further capitalise resonance enhancement effects, providing a more complete assignment of the species formed at the different stages of the MTO reaction, and highlighting how the use of different wavelengths is essential for obtaining the full mechanistic picture of this particular reaction. This is indeed one of the main differences with the work mentioned by the referee (Signorile et al., Catal Today 2019), as it provides more limited spectral assignments and thereby, very limited mechanistic information. Furthermore, it is important to note that - beyond differences in the type of catalyst material used (i.e. SAPO-34 vs. SSZ-13), the article of Signorile et al. is actually focused on the comparison of different reactor configurations, and not different wavelengths (or obtaining actual mechanistic information), which underscores the novelty of our work.

In addition, we would like to emphasise that the submitted manuscript actually includes references to some primary work published by Signorile et al. on this topic, although not the specific paper pointed out by the reviewer which comprises a follow up of their primary work, to compare different reactor configurations/setups to uncover species detected due to spatiotemporal differences in the reaction. This work was specifically referenced due to their efforts to avoid sample damage in our introduction section under references (now) 26 (relating to their work using a fluidised bed reactor) and (now) 35 (relating to their work on the spinning pellet reactor). In order to complete the set of references by Signorile et al. we have now further added a reference to the review mentioned by reviewer 2 (now reference 34).

The final paragraph of the introduction has been further edited to include a better explanation of the aim of the paper:

"We used a combination spectroscopy and molecular simulations to identify and intermediates and rationalise their behaviour in the reaction. By the application of Kerr-gated Raman spectroscopy with an excitation wavelength of 400 nm, it was possible to remove sample background fluorescence and simultaneously take advantage of resonance enhancement effects to obtain strong signals from hydrocarbon pool species, which are known to absorb light around this same wavelength.14,16–21 This was a rare opportunity to use a visible wavelength probe for Raman measurements on a system which is highly prone to fluorescence, allowed by the application of the Kerr-gated Raman spectrometer which allowed fluorescence rejection on a picosecond timescale.41 For a comparison, in this paper we show data collected during a repeated reaction of MTH over H-SSZ-13 catalyst using a Raman probe of 267 nm instead, allowing a direct comparison of signals recorded with the UV Raman probe against the visible wavelength probe (400 nm).

Comment 2. In their manuscript the authors stress the importance of resonance conditions. To this end, however, no experimental support (that is UV-Vis spectra) is provided. In principle, a rigorous discussion would require the recordance of operando UV-Vis spectra to reflect the current state of the catalyst.

Response. We would like to thank the reviewer for this suggestion. We actually agree that this would strengthen the paper, and have therefore added complementary operando UV-Vis data that shows the changing UV-Vis absorbance spectra and supports the importance of selective Raman excitation, in Figure S1 of the ESI.

Comment 3. Unfortunately, detailed catalytic (conversion, selectivity) and Raman spectroscopic (objective, laser intensity, spectral resolution) conditions are not reported in the manuscript.

Response. While we agree with the reviewer that quantitative catalytic data would be of interest, this is certainly limited by the time resolution required for the operando Raman experiments. In our studies, a mass spectrometer was used because this allows a faster sampling method (about 20 min for a gas chromatograph and 25 s for our mass spectrometer), which is required to be able to correlate, in a time-resolved manner, the adsorbed species formed on the catalyst surface (as seen by Raman) with the outlet products (mass spectrometer). An intrinsic limitation of mass spectrometry however, is the difficulty of completely decoupling contributions from multiple products, such as olefins and paraffins in the MTO reaction (see table below), and thereby to provide quantitative data (e.g. conversion and selectivity). We must note however, that the semi-quantitative trends observed by mass spectrometry and the reaction conditions employed in this work are comparable to those reported by leading catalytic groups reporting operando data in MTH (Signorile et al., Catal Today 2019, Signorile et al., Phys. Chem. Chem. Phys. 2018, Wragg et al., Angew. Chem. Int. Ed. 2012, and as described and referenced in our previous work (Lezcano et al., Nat. Mater. 2020).

Compound	Mass trace					
	25	26	27	41	42	43
Ethane	5	24	34			
Ethene	8	54	63			
Propane	2	10	44	13	8	23
Propene	4	11	39	100	70	4

In addition, we find it necessary to point out that the Raman data was recorded using a unique facility-built Raman setup, and not a commercial Raman microscope. Therefore, not all details requested by the reviewer apply to the spectrometer used in this work (e.g. a series of lenses and mirrors were used to focus the sample). To further clarify this, we have now expanded and edited the corresponding experimental section, which reads as follows:

“The new UV Raman data reported in this paper was acquired on the same custom-built spectrometer with some modifications as was used in ref 40 at the ULTRA facility, RAL UK,41 using the 3rd harmonic of the picosecond Ti-Sapphire laser to generate the 267 nm wavelength probe. The laser was operated at 10 kHz repetition rate, producing picosecond pulses (2 ps pulse length) at 800 nm fundamental wavelength. The 267 nm probe beam power at the sample was attenuated to 1 mW which corresponds to 100 nJ pulse energy. In contrast to what was reported in 40, for the present UV Raman the experiment was performed with the Kerr gate removed from the setup, and all the glass signal collection optics and visible-range mirrors replaced with the fused silica lenses and UV mirrors respectively. The collected signal beam was dispersed with a diffraction-grating spectrograph and detected with a UV-enhanced CCD. The data was accumulated for a total of 60 s. Raman measurements were taken at 10 °C intervals during the MTH reaction. The Linkam stage was attached to a raster system to move the sample in the plane perpendicular to the beam to avoid laser damage during the measurements. The laser spot size at sample was approx. 150 µm and it was rastered across 2 x 2 mm area following Lissajous pattern. This way the area irradiated by the laser was expanded significantly and the sample was irradiated in a near random pattern, and the effectiveness of the raster in preventing sample damage is illustrated in Figure S4.”

Comment 4. In particular, as no fluidized bed is used, detailed experimental conditions for the use of UV Raman spectroscopy need to be provided.

Response. As indicated above, we have now expanded and edited the corresponding experimental section. We would like however, to provide further clarification regarding the method used to avoid beam damage. As indicated in the both the new and the previous experimental section, rastering of the sample (developed at the ULTRA facility) was employed to avoid beam-induced damage (note reference to the rastering of the sample under the beam has been further expanded to read:

“The Linkam stage was attached to a raster system to move the sample in the plane perpendicular to the beam to avoid laser damage during the measurements. The laser spot size at sample was approx. 150 µm and it was rastered across 2 x 2 mm area following Lissajous pattern. This way the area irradiated by the laser was expanded significantly and the sample was irradiated in a near random pattern, and the effectiveness of the raster in preventing sample damage is illustrated in Figure S4.”

Please also find added to the SI, and shown below, an example of a measurement of toluene with and without the rastering, during our experiment with the Kerr-gated Raman spectrometer and 400 nm wavelength probe. where sample degradation is quickly observed without raster while the raster system switched on allows clear measurement of the spectrum of toluene which does not change upon repeating the measurement 4 times. We found the raster system to be excellent in helping to avoid beam-induced damage, in lieu of using a fluidised bed.

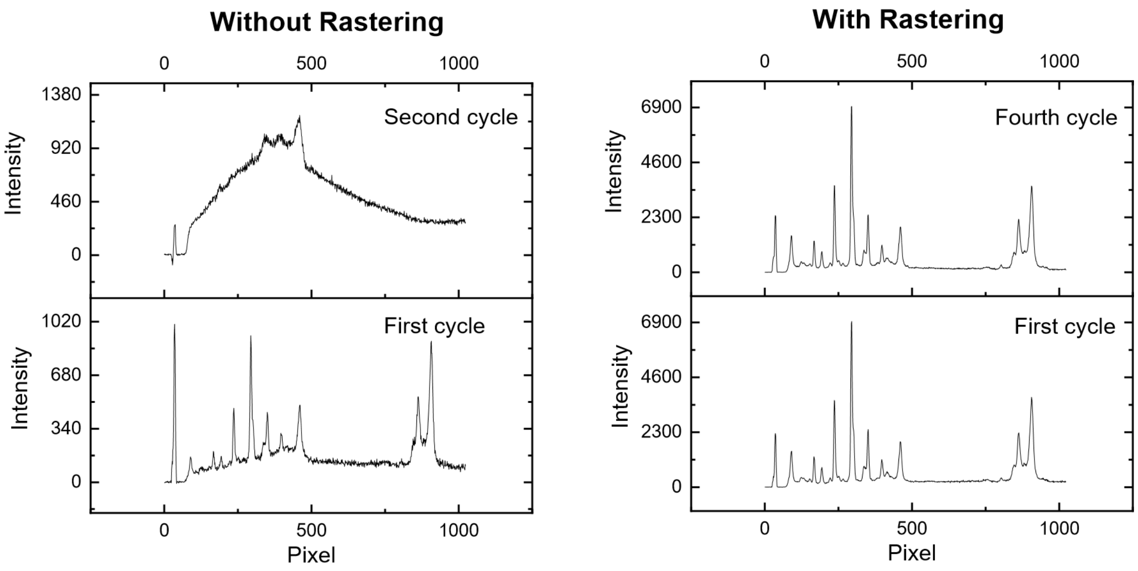

Comment 5. To the best of my knowledge, the manuscript does not fit the J Phys Chem Letter format. The manuscript may be considered for publication in J Phys Chem C subject to further review. When preparing a revised version, the authors should update the cited literature regarding regular as well as review articles containing UV-Raman spectroscopy.

Response. We consider that the cited literature is up-to-date and appropriate to support the work presented in the manuscript (e.g. see response to comment 1 regarding the work of Signorile et al.). Nevertheless, we have now extended the number of references containing UV Raman spectroscopy including references 2, 3, 5, 9, 12 and 13

Editor's comments

1) Please submit your publication files without any markups. Please include annotated version(s) of your revised publication file(s) with colored text or highlights indicating the revisions that you have made as "Supporting Information for Review Only."

2) Title: In both the main manuscript file and the Supporting Information, set the title in title case, with the first letter of each principal word capitalized.

3) Title: Using acronyms in title is discouraged. Please check for any acronyms in the title of the manuscript and Supporting Information and spell them out unless they are standard for the field.

3) Title and Author Lists: Title, author names, and affiliations must match in three places: (1) manuscript file, (2) supporting information, and (3) ACS Paragon Plus. Some author names are not spelled the same.

4) TOC Graphic: Please resize the TOC graphic per journal guidelines (2 in x 2 in).

5) Headers: Remove the section heading(s) throughout the body of the manuscript (you can leave Methods, Abstract, and TOC Graphic headings).

6) References: In both the main file and the supporting information, fix the style of all references to use JPCL formatting (check all references carefully). ***JPC Letters reference formatting requires that journal references should contain: () around numbers; author names; article title (titles entirely in title case or entirely in lower case); abbreviated journal title (italicized); year (bolded); volume (italicized); and pages (first-last). Book references should contain author names; book title (in the same pattern); publisher; city; and year. Websites must include date of access.

7) References: URLs are not preferred references because website content can be modified and, consequently, the reference information may lack permanence.

8) Supporting Information Statement: A brief, nonsentence description of the actual contents of each supporting information file is required. This description should be labeled Supporting Information and should appear before the Acknowledgement and Reference sections. Examples of sufficient and insufficient descriptions are as follows:

*Examples of sufficient descriptions: "Supporting Information: 1H NMR spectra for all compounds" or "Additional experimental details, materials, and methods, including photographs of experimental setup".

*Examples of insufficient descriptions: "Supporting Information: Figures S1-S3" or "Additional figures as mentioned in the text".

9) Supporting Information: Please number SI pages in the following format: "S1, S2…"

Yours faithfully,

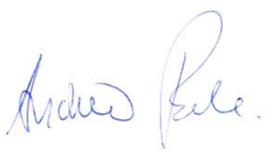

Andrew M. Beale
